# Supplementary material for: Two-dimensional materials-based probabilistic synapses and reconfigurable neurons for measuring inference uncertainty using Bayesian neural networks
Source: Nat Commun. 2022 Oct 17;13:6139. doi: 10.1038/s41467-022-33699-7 (PMC9576759; doi:10.1038/s41467-022-33699-7)
Supplement: Supplementary file 1 — Supplementary Information [file 41467_2022_33699_MOESM1_ESM.pdf]

Supplementary Information

Two-dimensional Materials-based Probabilistic Synapses and Reconfigurable Neurons for Measuring Inference Uncertainty Using Bayesian Neural Networks

Amritanand Sebastian[1,], Rahul Pendurthi[1], Azimkhan Kozhakhmetov[2], Nicholas Trainor[2,3], Joshua A. Robinson,[2,3] Joan M. Redwing[2,3],& Saptarshi Das[1,2,3,4,*]*

[1]*Deparment of Engineering Science and Mechanics, Penn State University, University Park, PA 16802*

[2]*Department of Materials Science and Engineering, Penn State University, University Park, PA 16802*

[3]*2D Crystal Consortium Materials Innovation Platform, The Penn State University, University Park, PA, 16802, US*

[4]*Department of Electrical Engineering and Computer Science, Penn State University, University Park, PA 16802*

Supplementary Figure 1

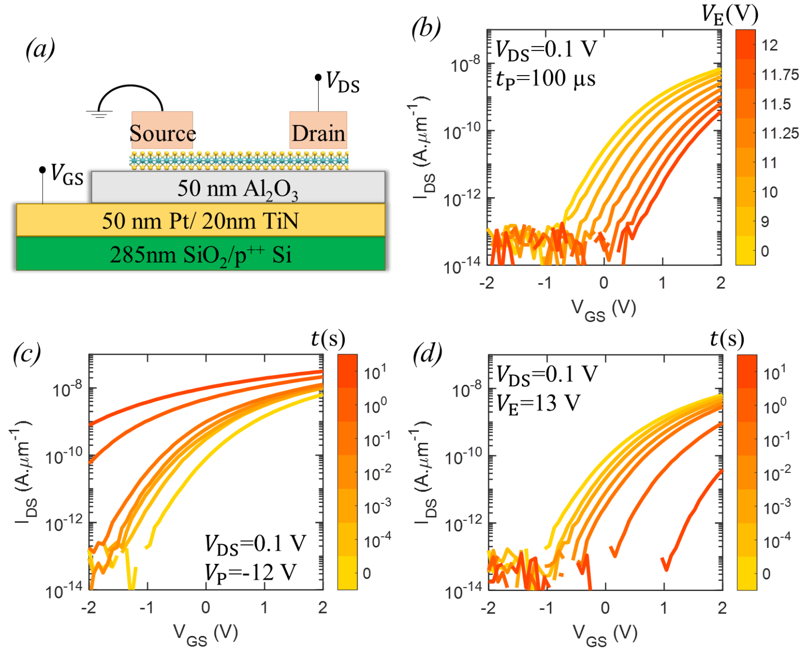

Supplementary Figure 1. Programmable MoS_2 memtransistor. *a) Schematic of a MoS_2 memtransistors with a local back-gated geometry, with a channel length and channel width of 5 µm. b) Transfer characteristics of post-erased MoS_2 memtransistor obtained by applying positive erasing voltages (V_E) of different amplitudes for pulse duration (t) of 100 µs. c) Transfer characteristics of post-programmed MoS_2 memtransistor by applying constant V_P for varying t. d) Transfer characteristics of post-erased MoS_2 memtransistor by applying constant V_E for varying t.*

Supplementary Figure 2

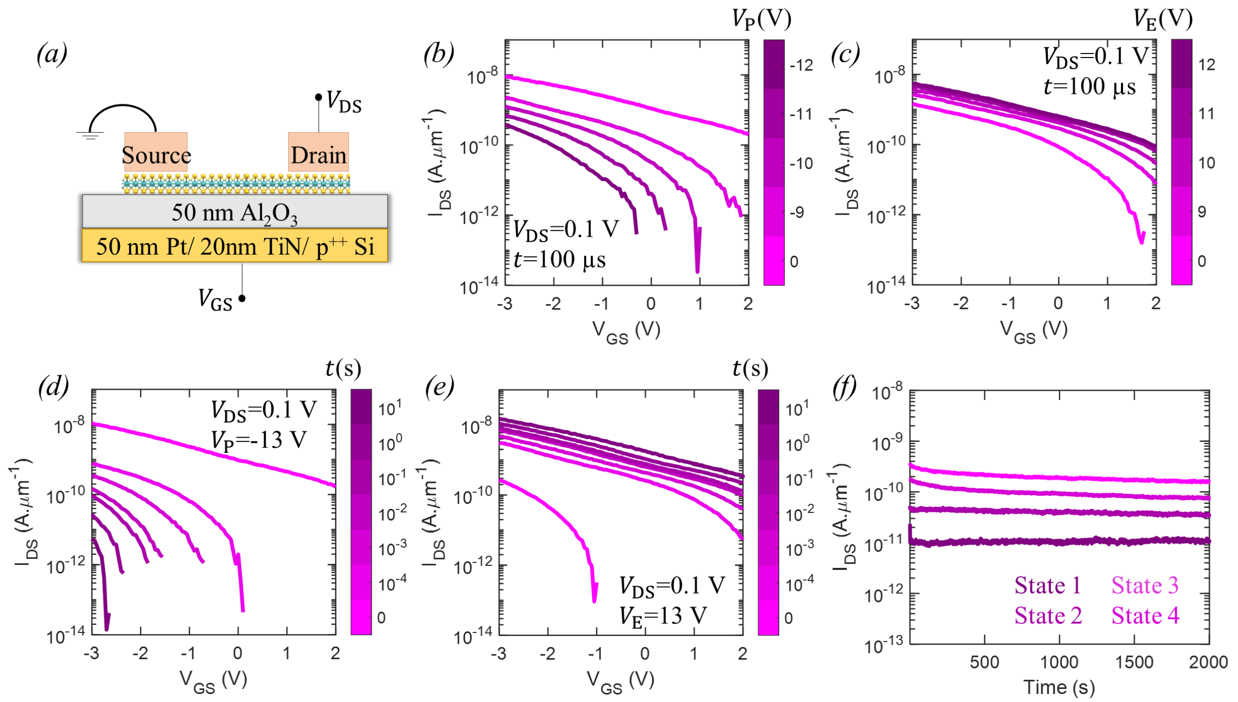

Supplementary Figure 2. Programmable WSe_2 memtransistor. *a) Schematic of a WSe_2 memtransistor with a back-gated geometry, with a channel length of 1 µm and channel width of 5 µm. b) Transfer characteristics of a post-programmed WSe_2 memtransistor obtained by applying V_P of different amplitudes for t of 100 µs. c) Transfer characteristics of post-erased WSe_2 memtransistor obtained by V_E of different amplitudes for t of 100 µs. d) Transfer characteristics of post-programmed WSe_2 memtransistor by applying constant V_P for varying t. e) Transfer characteristics of post-erased WSe_2 memtransistor by applying constant V_E for varying t. f) Analog retention characteristics, i.e., post-programmed I_{DS} versus time measured at V_{GS} = 0 V, for 4 different states.*

Supplementary Figure 3

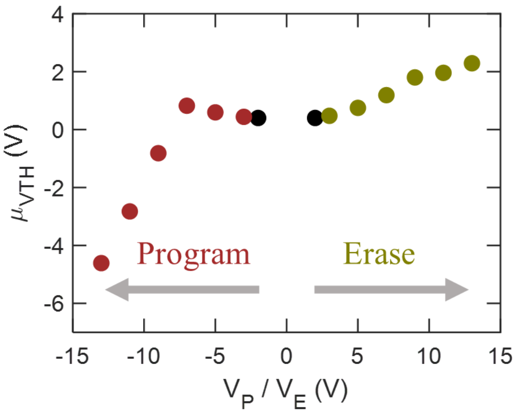

Supplementary Figure 3. *Mean threshold voltage ($\mu_{V_{TH}}$) as a function of V_P and erasing voltage V_E applied during V_{GS} sweeps. A significant increase in $\mu_{V_{TH}}$ is only seen for high V_P/V_E.*

Supplementary Figure 4

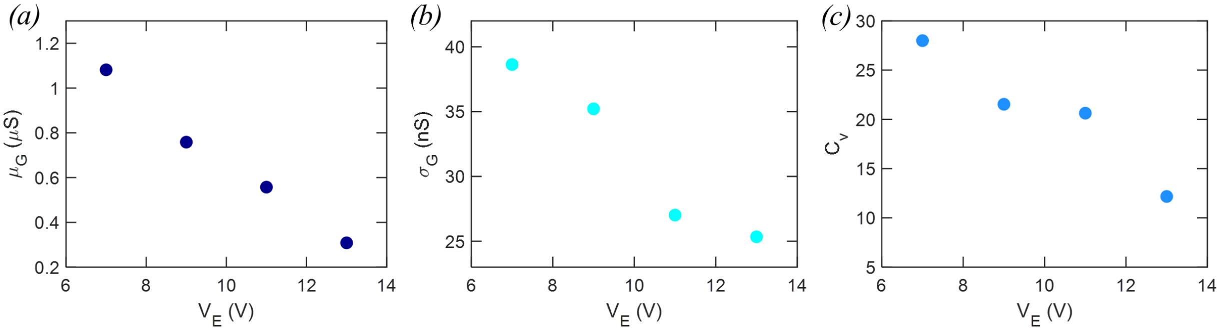

Supplementary Figure 3. Dependence of the Gaussian random number generation on V_E. *Dependence of a) μ_G, b) σ_G, c) C_v on V_E. Here, V_E is changed in the erase-program-read pulse cycle. μ_G and σ_G are seen to be coupled.*

Supplementary Figure 5

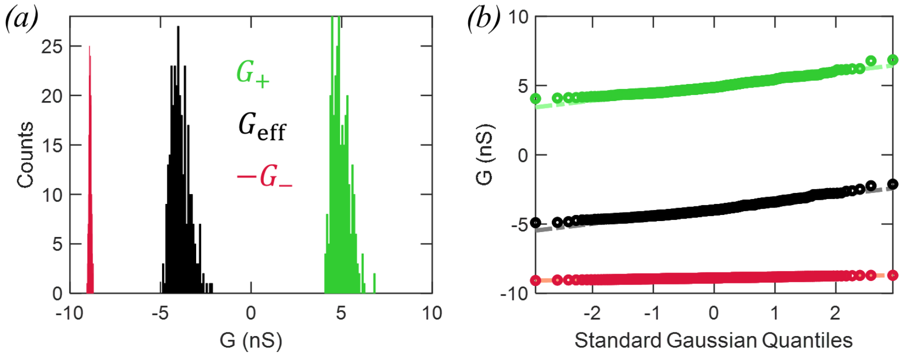

Supplementary Figure 5. Distributions in of conductance for the GRNG-based synapse. *a) Histogram demonstrating that G_+ and G_{eff} follows a Gaussian distribution, while G_- is almost constant. b) The quantile-quantile (Q-Q) plot of G_+, G_{eff}, and G_- confirming that G_+ and G_{eff} follow Gaussian distributions with similar σ, represented by similar slopes. G_- has a very flat line showing that its mostly constant.*

Supplementary Figure 6

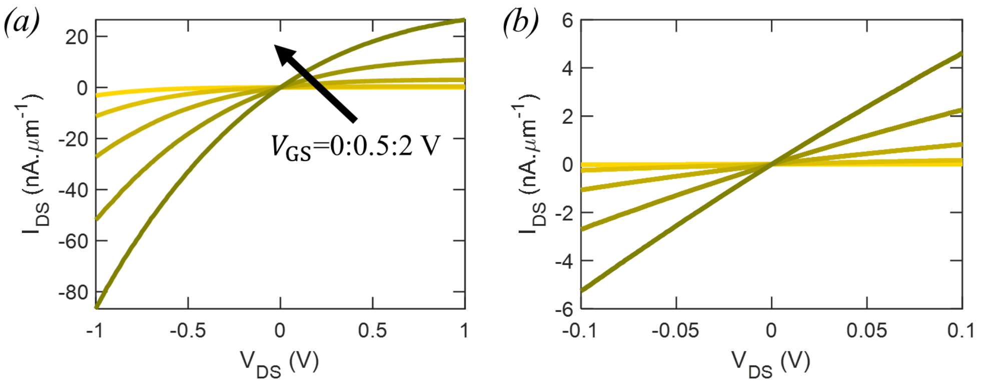

Supplementary Figure 6. Output characteristics of MoS_2 memtransistor. *The output characteristics of an MoS_2 for a) high drain voltages and b) low drain voltages. Within drain voltages of ±0.1 V, the MoS_2 is sufficiently linear and symmetric.*

Supplementary Figure 7

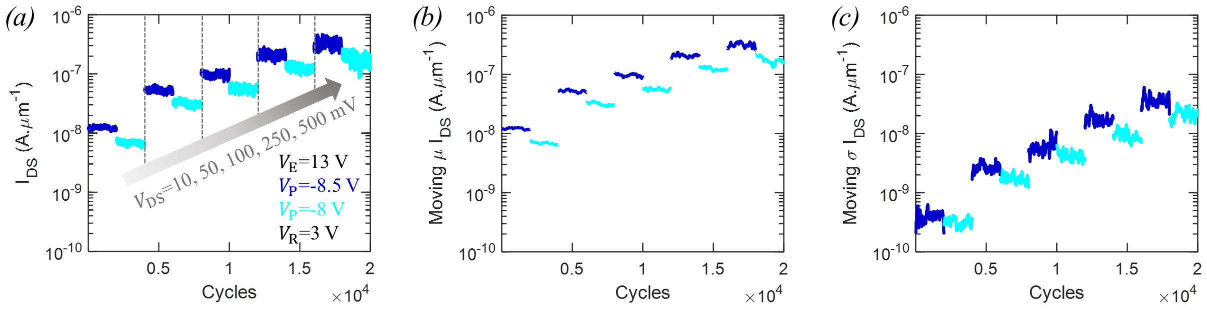

Supplementary Figure 7. Stability of random number generation. *a) Endurance characteristics of an* MoS_2 *memtransistor demonstrating various conductance states for a total of 20,000 cycles. The gate is subjected to successive erase-program-read pulses, and hence each cycle shown here includes the effect of a program and erase operation. Here, multiple current levels consisting of high and low current states (achieved with programming voltages,* V_P *of -8.5 V and -8 V, respectively) for different* V_{DS} *are demonstrated. b) Moving mean and c) moving standard deviation for these different states. The moving mean and standard deviation are obtained across 100 samples at a time.*

Supplementary Figure 8

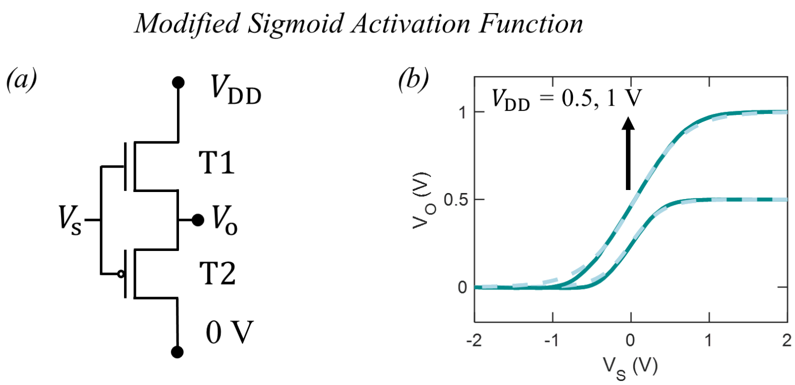

Supplementary Figure 8. Modified sigmoid activation function. *a) Schematic of circuit for the modified sigmoid activation function using a n-type MoS_2 memtransistor ($T1$) and a V-doped p-type WSe_2 memtransistor ($T2$), where the input voltage (V_S) is applied to the gate terminal of $T1$ and $T2$. b) The transfer characteristics of the circuit (solid line) i.e., output voltage (V_O) versus V_S, closely models the sigmoid activation function (dotted line).*

Supplementary Note 1

Supplementary Fig. 9a demonstrates the device-to-device variation across 40 MoS_2 memtransistors while performing program/erase operation. Here, the progressively higher programming voltage pulses (V_{P}) followed by progressively higher erase voltage pulses (V_{E}) are applied to the MoS_2 memtransistors and the drain-to-source current (I_{DS}) is measured at a read voltage (V_{R}) of 0 V. This is repeated for 2 cycles, to demonstrate cycle-to-cycle variation. While the cycle-to-cycle variation is beneficial for a BNN, device-to-device variation is detrimental to its operation. Supplementary Fig. 9b and Supplementary Fig. 9c demonstrates the relationship between the change in current (ΔI_{DS}) as a result of erase or program operation and the starting current ($I_{\mathrm{DS,start}}$) before each erase and program operation, respectively. They follow a power law relationship given by Eq. S1.

$$\Delta I_{\mathrm{DS}} = p * {I_{\mathrm{DS,start}}}^{q} \qquad \text{[S1]}$$

Here, p is the scaling factor and q is the exponential factor. If we account for this dependence, the device-to-device variation can be reduced. $\Delta I_{\mathrm{DS}}/{I_{\mathrm{DS,start}}}^{q}$ follows a Gaussian distribution, as shown representatively in Supplementary Fig. 9d for the erase operation.

Supplementary Figure 9

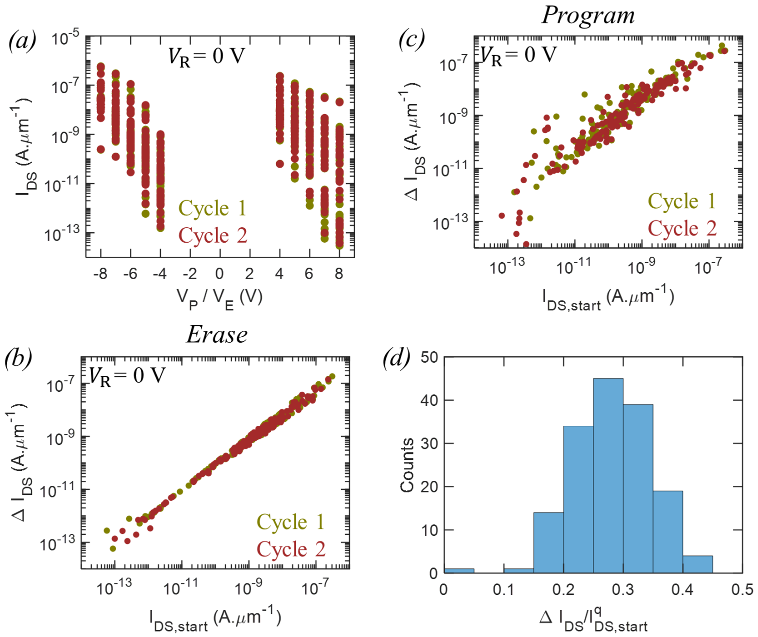

Supplementary Figure 9. Modelling Variation. *a) Device-to-device variation across 40 MoS_2 memtransistors for different program and erase voltages. Relationship between the change in the drain current (ΔI_{DS}) as a result of b) erase and c) program operation and the starting current ($I_{DS,start}$). e) Histogram of $\Delta I_{DS}/I_{DS,start}{}^q$ for the erase operation, following a Gaussian distribution.*

Supplementary Table 1

Supplementary Table 1. Memory Technologies Energy Benchmark				
	Memristor	PCM	NAND	2D Memtransistor
Cell Elements	1T1R	1T1R	1T	1T
Read Time (ns)	<50	<60	<50	<50
Read Voltage (V)	<3	3	2	0
Program/Erase Time (ns)	<250	60	10^6	10^5
Program/Erase Voltage (V)	<3	3	15	13
Program/Erase Energy (fJ)	<50	6×10^3	10	10
